# A Rare Case of Atypical Pleomorphic Neoplasm of Pineal Region in a Child: A Case Report

**DOI:** 10.7759/cureus.10515

**Published:** 2020-09-17

**Authors:** Saurabh Kataria, Karun Neupane, Zahoor Ahmed, Erum Noor, Usama Rehman

**Affiliations:** 1 Neurology and Neurocritical Care, University of Missouri Columbia, Columbia, USA; 2 Internal Medicine, Manipal College of Medical Sciences, Pokhara, NPL; 3 Internal Medicine, King Edward Medical University, Lahore, PAK; 4 Internal Medicine, Jinnah Medical and Dental College, Karachi, PAK; 5 Anesthesiology, Mayo Hospital, Lahore, PAK

**Keywords:** atypical pleomorphic neoplasm, pineal gland, oncology

## Abstract

A 10-year-old boy with no past medical history presented with complaints of nausea and vomiting associated with morning headache for the last month. Ophthalmic nerve and eye exam showed diplopia and strabismus with no other significant findings on physical and neurological examination. Magnetic resonance imaging (MRI) of the brain revealed a homogenous hyperdense and enhancing mass in the pineal region. The endoscopic biopsy of the pineal region demonstrated the cells with highly pleomorphic and hyperchromatic nuclei with an increase in mitotic activity. There were many vessels but no area of vascular proliferation and necrosis. Granular bodies with eosinophilia were identified. Immunohistochemistry was positive for class III b-tubulin with epidermal growth factor receptor (EGFR) staining and glial fibrillary acidic protein (GFAP). Immunostaining was positive for p53, Phosphatase and Tensin homolog (PTEN), and oligodendrocyte transcription factor (OLIG2), while staining for cluster of differentiation (CD)34, cytokeratin (CK), human melanoma black (HMB)45, and isocitrate dehydrogenase (IDH)-R132H mutation was negative, consistent with atypical pleomorphic neoplasm of the pineal region. The patient underwent tumor resection via a sub-occipital trans-tentorial approach, followed by one dose of chemotherapy. The patient experienced a resolution of the symptom and was doing well on his bi-monthly follow up.

## Introduction

Pineal gland tumors contribute to less than 1% of all the primary brain tumors in Europe and North America. They are more common in Asian countries and among the one- to 12-year age group (3% of brain tumors) [1,2].

The most common tumors in the pineal gland include germ cell tumors, gliomas, and pineal parenchymal tumors [3]. The World Health Organization (WHO) has classified pineal gland tumors into four groups: pineocytoma (grade I), pineal parenchymal tumors of intermediate differentiation (grade II or III), the papillary tumor of the pineal region (grade II or III), and pineoblastoma (grade IV) [4]. Pleomorphic pineal gland tumors are exceptionally uncommon, with only eight reported cases in the literature [5]. Herein, we present a case of a child with an atypical pleomorphic neoplasm of the pineal gland.

## Case presentation

A 10-year-old boy with no past medical history was brought by his parents in the outpatient clinic, with complaints of nausea and vomiting for two days. He also had a headache for the last month. The pain was dull, localized to the scalp, and the occipital region occurring usually in the morning and lasted for a few hours. The pain was relieved by taking an analgesic. He was in the normal state of health before one month. On initial evaluation, the temperature was 37°C, blood pressure was 120/70 mmHg, heart rate was 91 beats per minute, respiratory rate was 20/minute, and oxygen saturation was 99% on room air. On physical examination, the patient was healthy and appeared oriented and alert with intact cognition. His skin, extremities, and pulses were normal. However, the patient was anxious with frequent blinking of eyes. On neurological examination, his power, and coordination, and gait were intact, and his toes were down going. The sensation was intact bilaterally, and the reflexes were intact on both sides of the body. His cranial nerve examination was also unremarkable except ophthalmic nerve and eye examination. The patient was noted to have strabismus and diplopia. There was no evident deformity on his face or eye, and he did not have any muscle weakness. He had no history of previous trauma or fall.

**Figure 1:**
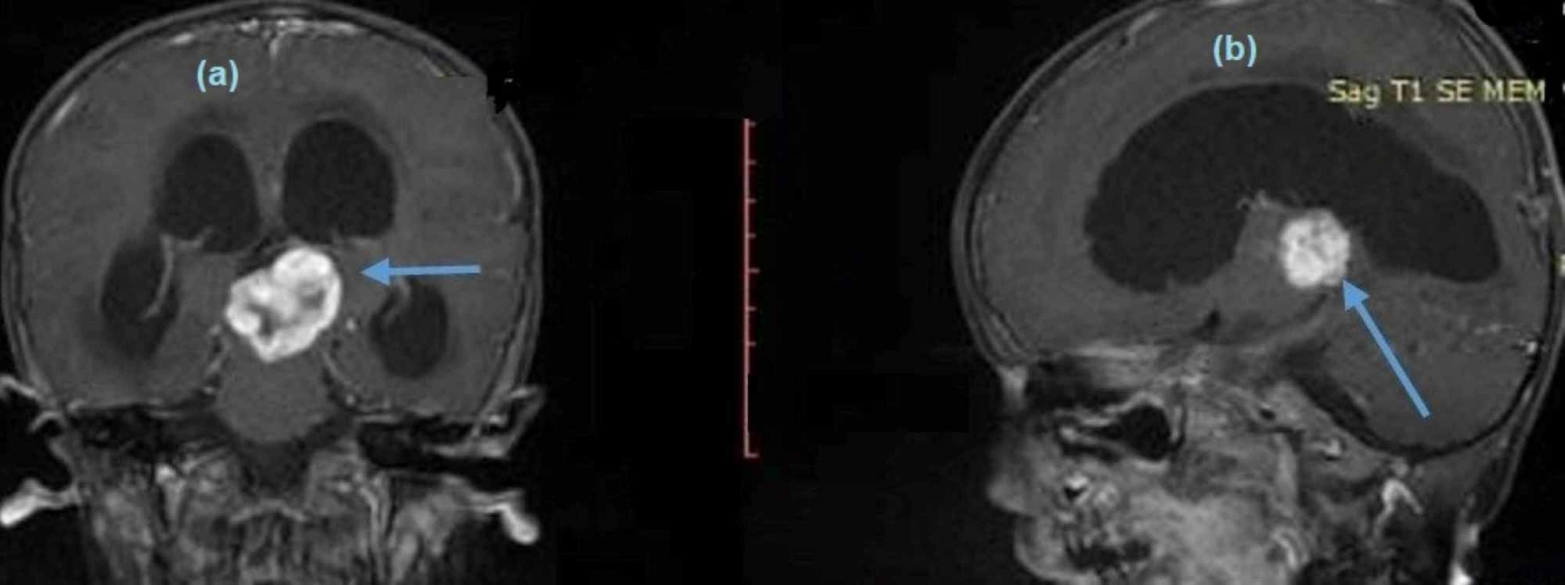
Magnetic resonance imaging (MRI) of the brain showing a homogenous hyperdense and enhancing mass in the pineal region in coronal (a) and sagittal (b) planes.

Imaging studies were performed, and the magnetic resonance imaging (MRI) of the brain revealed a homogenous hyperdense and enhancing mass in the pineal region (Figure 1).

The patient underwent endoscopic biopsy of the pineal region, which demonstrated cells with highly pleomorphic and hyperchromatic nuclei. A significant increase in mitotic activity was detected. There were many vessels but no area of vascular proliferation and necrosis. Granular bodies with eosinophilia were identified. Immunohistochemistry was positive for class III b-tubulin with epidermal growth factor receptor staining (EGFR) and glial fibrillary acidic protein (GFAP). Immunostaining was positive for p53, Phosphatase and Tensin homolog (PTEN), and oligodendrocyte transcription factor (OLIG2), while staining for cluster of differentiation (CD)34, cytokeratin (CK), human melanoma black (HMB)45, and isocitrate dehydrogenase (IDH)-R132H mutation was negative. That was consistent with the atypical pleomorphic neoplasm of the pineal region.

The patient underwent tumor resection via a sub-occipital trans-tentorial approach, followed by one dose of chemotherapy. The patient experienced a resolution of the symptom and was doing well on his bi-monthly follow up. 

## Discussion

Atypical pleomorphic neoplasms of pineal glands are usually classified as either pleomorphic xanthoastrocytoma (PXA) or pleomorphic granular cell astrocytoma (PGCA) [5]. The presenting symptoms of these tumors include headache, nausea, and vomiting. These have been attributed to hydrocephalus secondary to compression of the aqueduct. Parinaud syndrome, gait disturbances, cranial nerve palsies, and seizures are other less common features [6,7].

The diagnosis of brain neoplasm is based on clinical presentation and the use of imaging modalities. The MRI characteristics of these tumors can differ and can include an enhancing lesion with calcification or a lesion with cystic and solid components, which appear hypodense on the T1 weighted image (T1WI) and heterogeneous in T2 weighted image (T2WI) [8,9]. PXA tumors histologically consist of pleomorphic cells with multinucleated giant cells with varying shape and size. These tumors are usually positive for GFAP immunoreactivity and are rich in reticulin network with reticulin stain [10].

PGCA has histology similar to PXA. The differences include the presence of a large number of mitochondria and the absence of reticulin fibers and a basement membrane between adjacent cells in PGCA. Coarse granular cells with d-periodic acid Schiff staining are other features of PGCA [11,12]. The tumor that does not strictly present as PXA or PGCA has been labeled as atypical by Nitta et al. and Praver et al. [5,11].

The treatment of pineal region tumors is based on the size, its histological nature, and the neurosurgeon’s habits. Sub-temporal, parieto-occipital, or trans-tentorial approaches are adopted for tumor resection. In advanced cases, tumor resection is followed by chemotherapy as adjuvant therapy [11]. Subtotal resection can also be justified in slowly growing tumors and in those patients where total resection was determined to be too risky at the time of surgery.

The case reported here has pleomorphic nuclei but lacks d-periodic acid Schiff staining, hence fitting as an atypical pleomorphic astrocytoma. Like other reported cases of pleomorphic pineal tumors, our patient here also underwent surgical resection (and one dose of chemotherapy) and experienced resolution of the symptoms suggesting that surgical excision is likely an adequate treatment in such cases.

## Conclusions

Strabismus could be a presenting sign of atypical pleomorphic neoplasm of the pineal region, though it is highly uncommon. Pineal region neoplasm should be considered as a differential diagnosis in those patients who present with a long-standing history of morning headache associated with diplopia and strabismus. Surgical resection followed by chemotherapy results in a favorable outcome and alone can yield excellent long-term results for the neoplasm falling within the spectrum of pleomorphic lesions of the pineal gland.
